# Therapeutic Potential Targeting Gut Microbiota Modulation With Emphasis on *Lactobacillus* spp. in Common Metabolic Disorders: A Systematic Review

**DOI:** 10.1155/sci5/3367875

**Published:** 2025-11-03

**Authors:** Md. Mizanur Rahaman, Phurpa Wangchuk, Subir Sarker

**Affiliations:** ^1^Biomedical Sciences and Molecular Biology, College of Medicine and Dentistry, James Cook University, Townsville 4811, Queensland, Australia; ^2^Australian Institute of Tropical Health and Medicine, James Cook University, Townsville 4811, Queensland, Australia; ^3^College of Science and Engineering, James Cook University, Nguma Bada Campus, McGregor Rd, Smithfield, Cairns 4878, Queensland, Australia; ^4^Australian Institute of Tropical Health and Medicine, James Cook University, Nguma Bada Campus, McGregor Rd, Smithfield, Cairns 4878, Queensland, Australia; ^5^Department of Microbiology, Anatomy, Physiology and Pharmacology, School of Agriculture, Biomedicine and Environment, La Trobe University, Melbourne, Victoria 3086, Australia

**Keywords:** dysbiosis, gut microbiome, inflammation, metabolic disorders, therapeutic potential

## Abstract

Metabolic disorders are complex conditions that arise from abnormal biochemical reactions, disrupting normal metabolic processes. The most prevalent metabolic disorders include obesity, Type 2 diabetes mellitus (T2DM), cardiovascular disease (CVD), nonalcoholic fatty liver disease (NAFLD), and inflammatory bowel disease (IBD). Despite extensive research, no definitive therapeutic strategy has been established for a complete cure. Emerging evidence suggests that gut microbiome dysbiosis plays a critical role in the pathogenesis of these disorders, as maintaining microbial homeostasis is essential for metabolic health. Short-chain fatty acids (SCFAs) are a key metabolite produced by gut microbiota and exhibit significant therapeutic potential by serving as an energy source for colonocytes, enhancing gut barrier integrity, and modulating inflammation. Our analysis reveals that targeted microbial modulation, particularly through SCFA-producing probiotics and prebiotics, consistently benefits host metabolism and reduces systemic inflammation across multiple conditions. This review highlights the importance of gut microbiota as a viable therapeutic target and underscore the need for further clinical trials to validate microbiome-based interventions in metabolic disease management.

## 1. Introduction

Metabolic disorders, including obesity, Type 2 diabetes mellitus (T2DM), cardiovascular disease (CVD), nonalcoholic fatty liver disease (NAFLD), and inflammatory bowel disease (IBD), represent a significant global health burden, contributing to rising morbidity and mortality rates. These disorders arise from disruptions in fundamental metabolic processes, leading to energy dysregulation and redox imbalance [1]. Key risk factors, collectively referred to as metabolic syndrome, include dyslipidemia, leptin and adiponectin dysregulation, insulin resistance, impaired insulin secretion, and glucose intolerance [2]. At the cellular level, metabolic disorders disrupt essential biochemical pathways involved in macronutrient metabolism, thereby impairing homeostasis [3]. The underlying pathophysiology is multifaceted, involving impaired insulin sensitivity, excessive visceral fat accumulation, lipid metabolism disturbances, and vascular endothelial impairment, hypertension, genetic predisposition, and a hypercoagulable state [4]. Despite significant advances in understanding disease mechanisms, effective long-term management remains a challenge due to the complex and multifactorial nature of these conditions. Current therapeutic strategies, such as pharmacological interventions and lifestyle modifications, often yield variable outcomes and are limited by poor patient adherence, side effects, and a lack of personalized treatment approaches [5, 6]. Although bariatric surgery has shown promise in severe cases, its accessibility and long-term sustainability remain concerns [7, 8].

Gut microbiome plays a pivotal role in the treatment of metabolic disorders, making it a promising candidate for therapeutic interventions. The adult gut microbiota contains 10–100 trillion microorganisms, which is equal to ten times the total amount of human somatic and germ cells [9]. This microbial ecosystem exerts profound effects on host physiology, influencing immune system development, gut epithelial homeostasis, pathogen defense, and drug metabolism [10–13]. Despite its vast diversity, gut microbiota is largely dominated by four major phyla, *Firmicutes*, *Bacteroidetes*, *Actinobacteria*, and *Proteobacteria*, with other phyla contributing to a lesser extent [14]. This microbiota influences host metabolism through various mechanisms, including the production of SCFAs, bile acid metabolism, regulation of gut permeability, and modulation of inflammatory pathways [15]. Given the increasing prevalence of metabolic disorders such as obesity and T2D, probiotics and prebiotics have gained considerable attention for their potential in modulating gut microbiota composition and function, particularly in pediatric populations [16–18]. Probiotic-mediated modulation of intestinal microbiota has been shown to promote microbial homeostasis, offering potential benefits as an adjunctive therapy for T2D and insulin resistance [18, 19]. Additionally, probiotic supplementation has demonstrated the ability to reduce inflammation in mesenteric adipose tissue (MAT), thereby mitigating hyperglycemia and insulin resistance [20, 21]. Furthermore, a recent study underscores the role of gut microbiota in metabolic dysfunction–associated fatty liver disease (MAFLD). Abenavoli et al. [22] reviewed therapeutic approaches of Mediterranean diet, probiotics, and fecal microbiota transplantation (FMT), highlighting their potential benefits while also indicating limitations in clinical studies, due to small sample sizes and short follow-up durations. However, challenges such as variability in microbiome responses, the long-term stability of microbial interventions, and the need for robust clinical validation must be overcome to maximize the therapeutic efficacy of gut microbiome in combating metabolic disorders.

This systematic review critically examines the role of gut microbiota in mitigating common metabolic disorders. By synthesizing findings from preclinical and clinical studies, we aim to delineate the therapeutic potential of targeting gut microbiota and identify key microbial signatures associated with metabolic health. Addressing the interaction between gut microbiota and the host's metabolic processes may pave the way for innovative, microbiome-based strategies to combat metabolic disorders and improve global health outcomes.

## 2. Methodology

### 2.1. Search Strategy

This systematic review was conducted following the guidelines set by the Preferred Reporting Items for Systematic Reviews and Meta-Analyses (PRISMA) 2020. A comprehensive literature search was carried out across Google Scholar, Web of Science, Scopus, and PubMed databases to locate relevant studies examining the relationship between gut microbiota and metabolic disorders, with a specific focus on the therapeutic role of *Lactobacillus* spp.

Search terms included “gut microbiota,” “metabolic disorders,” “obesity,” “T2DM,” “CVD,” “NAFLD,” “IBD,” “probiotics,” “prebiotics,” “dietary modifications,” “*Lactobacillus* spp.,” “SCFAs,” “biomarkers” “therapeutic applications,” “mechanism,” and “clinical evidence.” Boolean operators (AND and OR) were used to expand the search strategy. Our search did not impose any language restrictions. The studies were thoroughly evaluated, and key information was extracted, culminating in an overall conclusion. The literature search covered relevant studies published from 2002 to January 2025. All methodologies, including search terms and inclusion criteria, were based on PRISMA guidelines. Additional materials such as search strategy, PRISMA checklist, and extracted data tables are available upon reasonable request to ensure full transparency and reproducibility.

### 2.2. Selection of Studies With Inclusion and Exclusion Criteria

Eligible studies for inclusion were as follows: (i) studies that were conducted in vitro, ex vivo, or in vivo, or using mammalian or human models; (ii) studies involving gut microbiome in relation to one or more of the following—obesity, T2DM, CVD, NAFLD, and IBD; (iii) studies that reported therapeutic interventions or applications (e.g., probiotics, prebiotics, dietary modifications); and (iv) studies that were primary research articles published in English.

Studies of the following nature were excluded: (i) duplicated data, titles, and/or abstracts that did not fulfill the inclusion criteria; (ii) articles published in languages other than English; (iii) case reports, letters, editorials, and commentaries; (iv) studies lacking full-text access; (v) studies that did not report the therapeutic activity against common metabolic disorders; and (vi) other metabolic disorders rather than obesity, T2DM, CVD, NAFLD, and IBD.

The selection of these five metabolic disorders was based on their high prevalence, strong association with gut microbial dysbiosis, and clinical significance. The exclusive focus on *Lactobacillus* spp. was justified by its growing prominence in the recent literature as a key probiotic species with demonstrated efficacy in modulating host metabolic functions.

### 2.3. Database Reports

**Figure 1:**
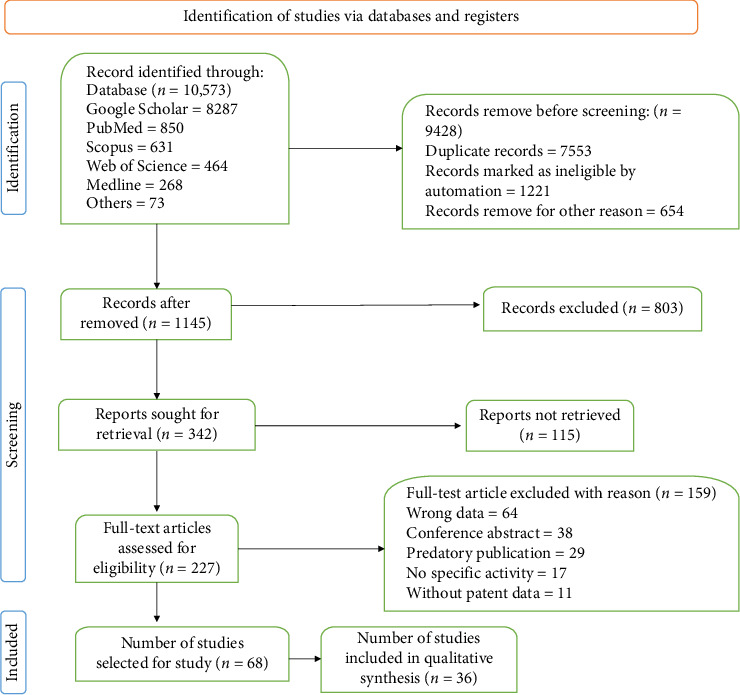
PRISMA flow diagram based on data extraction.

A total of 10,573 scientific articles were retrieved from various databases as of January, 2025. Subsequently, 97.85% of the articles were excluded due to duplication, irrelevance, insufficient data, or automation systems classifying them as unsuitable. Following the inclusion criteria, 227 articles on microbiome and metabolic disorders were identified with desired information. Letter 68 article selected for further study. Final 36 article included for qualitative synthesis. A PRISMA flow diagram illustrating the data collection process for microbiome and metabolic disorders is presented in Figure 1. A purposive sampling strategy was adopted to ensure the selection of studies with high methodological quality and clinical relevance. Reference management was conducted using EndNote, and data were extracted independently by two authors. Disagreements were resolved through discussion to reduce selection bias and maintain consistency.

### 2.4. Risk of Bias Assessment

**Figure 2:**
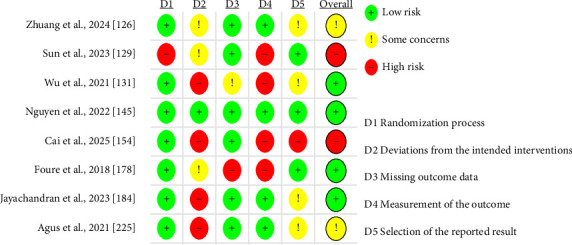
Risk-of-bias assessment. According to the Cochrane risk-of-bias tool for randomized trials (RoB 2) interventions, +: low risk of bias; −: high risk of bias; !: some concern risk of bias.

To assess the reliability of results from selected studies, we used Cochrane risk-of-bias assessment criteria, specifically the RoB 2 tool version, dated August 22, 2019. This tool evaluates risk across several domains: randomization process, deviations from intended interventions, missing outcome data, outcome measurement, selection of reported results, and other potential sources of bias. Each category comprised questions across these domains, with responses categorized as “YES” (indicating low risk of bias, color-coded green), “NO” (indicating high risk of bias, color-coded red), or “Some Concern” (indicating uncertain risk of bias, color-coded yellow) (Figure 2). In addition to the summary figure (Figure 2), per-study risk-of-bias tables were prepared for each included study to provide a detailed evaluation (see Supporting Table S3). No formal assessment of reporting bias or certainty of evidence (GRADE) was performed, as the review aimed to qualitatively synthesize findings without statistical pooling.

### 2.5. Data Items

We sought data on the following primary outcomes: (i) metabolic outcomes (body weight, BMI, lipid profile, fasting glucose, HbA1c), (ii) inflammatory markers (CRP, IL-6, TNF-α), (iii) gut microbiota composition (relative abundance of key taxa, diversity indices), and (iv) SCFA concentrations. Secondary variables included study population characteristics (species, age, sex, health status), intervention type (probiotic, prebiotic, dietary modification), dose and duration, comparator, study design, funding source, and country of study. Missing or unclear data were recorded as “NR” (not reported) (see Supporting Table S1).

### 2.6. Synthesis Methods

As no meta-analysis was conducted, no statistical effect measures (risk ratios, mean differences) were calculated; the results are presented narratively. Studies were grouped by the type of metabolic disorder (obesity, T2DM, NAFLD, CVD, IBD) and intervention category (probiotic, prebiotic, dietary modification). Missing or unclear data were noted as “NR,” and no imputation was performed. No formal heterogeneity or sensitivity analyses were undertaken due to the qualitative nature of the synthesis.

### 2.7. Ethical Statement

This systematic review did not involve direct experimentation on human or animal subjects and therefore did not require ethical approval. However, all original research articles included in this review reported approval from their respective institutional ethics committees. No identifiable patient data were used, and the review strictly adhered to PRISMA guidelines for transparency and reproducibility.

### 2.8. Review Registration

This review was not registered in PROSPERO or any other database, and no formal protocol was prepared prior to commencement. This was due to time constraints and the narrative synthesis approach adopted.

## 3. Results and Discussion

### 3.1. Common Metabolic Disorders

Metabolic disorders encompass a broad spectrum of conditions that disrupt the body's normal metabolic processes, which are essential for the conversion of nutrients into energy and synthesis of vital molecules. These disorders can affect the metabolism of carbohydrates, fats, proteins, and other crucial substances, leading to either the overproduction or underproduction of metabolic products. Such imbalances manifest in a range of symptoms and contribute to various health complications. Recent studies highlight the significant role of gut microbiome in influencing metabolic health. Dysbiosis, or imbalance in gut microbial composition, has been linked to disturbances in metabolic pathways, including insulin resistance, altered lipid metabolism, and chronic inflammation. A comprehensive overview of these disorders, including their pathophysiology, associated microbiome alterations, and clinical relevance, is summarized in Table 1.

#### 3.1.1. Obesity

**Figure 3:**
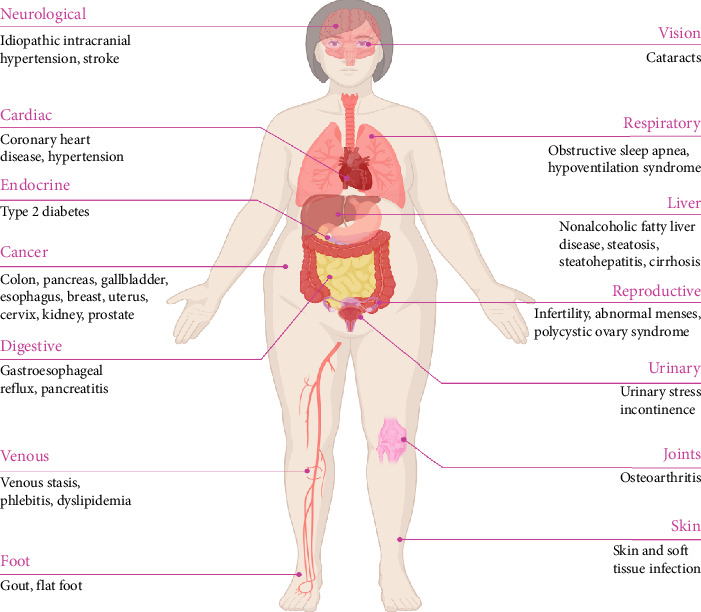
Schematic representation of obesity-associated complications in the human body. Obesity contributes to a wide spectrum of systemic disorders, including cardiovascular diseases (hypertension, atherosclerosis, and heart failure), metabolic dysfunctions (T2D and NAFLD), respiratory impairments (obstructive sleep apnea), and musculoskeletal conditions (osteoarthritis). Moreover, obesity is linked to an increased risk of certain cancers, neurodegenerative disorders, and immune dysfunction. Several of these complications, particularly cardiovascular and metabolic disorders, are life-threatening. Created in https://BioRender.com.

Obesity is a chronic, multifactorial, and relapsing noncommunicable disease characterized by an excessive or abnormal accumulation of body fat, which significantly increases health risks [37]. It is widely recognized as a major contributing factor to the development of both noncommunicable and communicable diseases [38–40]. Since 1999, the prevalence of obesity among U.S. adults, defined as a body mass index (BMI) of 30 or greater, has risen from 30% to 42%, with projections indicating that nearly 50% of adults will be affected by 2030 [41]. Obesity affects 19% of women and 14% of men globally [42]. It primarily results from a chronic energy imbalance, where excessive caloric intake from food and beverages and not enough energy expenditure through physical activity; however, additional factors such as microbiome imbalances, genetic predisposition, health disparities, environmental influences, and commercial determinants also play a role in the development of overweight and obesity [43, 44]. Obesity can influence the gut microbiota both structurally and functionally [24], while the gut microbiota, in turn, plays a role in regulating nutritional status [45–47]. An increased abundance and diversity of specific bacterial populations may contribute to enhanced energy storage and metabolic processes, ultimately leading to obesity [24, 48]. Obesity significantly increases the risk of various health complications (Figure 3), with evidence indicating that they are major contributors to noncommunicable diseases [49, 50]. For instance, obesity is associated with increased risks of cardiovascular issues, including obstructive sleep apnea, T2D, irregular menstruation in adolescent girls, and increased cholesterol levels [51, 52].

#### 3.1.2. T2D

**Figure 4:**
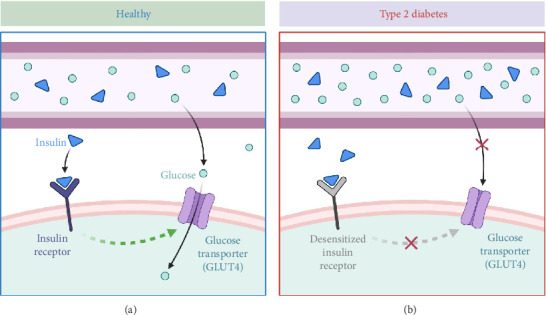
Comparison of glucose uptake in healthy individuals versus those with Type II diabetes. (a) A healthy state where insulin binds to its receptor, triggering the activation of glucose transporter (GLUT4) to facilitate glucose uptake into the cell. (b) Type II diabetes, where insulin receptors become desensitized, preventing GLUT4 activation and leading to impaired glucose uptake, resulting in elevated blood glucose levels. Created in https://BioRender.com.

The global burden of T2DM among older adults is increasing rapidly, largely due to longer life expectancy and prolonged exposure to cardiometabolic risk factors including excess body fat, muscle loss, and decreased physical activity [53–57]. In the time span from 2017 and 2045, the number of adults aged 60 years and older with diabetes approximately rises from 122 million to 253 million, paralleling the growth of global population of adults aged 65–99 years from 652 million to 1.42 billion [58]. T2DM is thought to result from the interplay of several risk factors, including elevated serum uric acid levels, inadequate sleep, smoking, depression, cardiovascular conditions, lipid imbalances, high blood pressure, aging, ethnic background, family history of diabetes, lack of physical activity, and obesity [59, 60]. The pathophysiology of T2DM involves insulin resistance and initial hyperinsulinemia, followed by a progressive decline in pancreatic β-cell function (Figure 4) [61, 62]. Furthermore, emerging studies have highlighted the relationship between gut microbiota and T2DM [63–66]; for example, one study identified 43 bacterial taxa showed significant differences between obese individuals with T2DM and healthy controls, indicating that *Acidaminococcales*, *Bacteroides plebeius*, and *Phascolarctobacterium* sp. CAG207 could serve as potential biomarkers for T2DM [67].

#### 3.1.3. NAFLD

**Figure 5:**
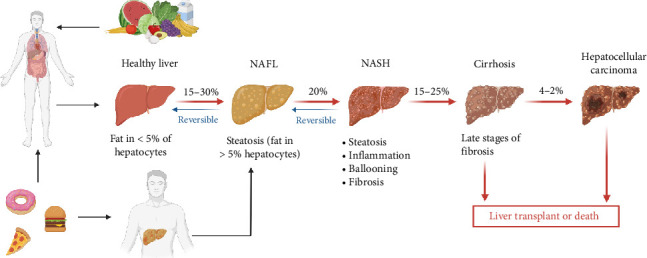
The progression of NAFLD from a healthy liver to hepatocellular carcinoma. The initial stages, NAFL (steatosis) and NASH (inflammation and fibrosis), are reversible with proper lifestyle changes. Cirrhosis and hepatocellular carcinoma are advanced stages requiring medical intervention, often leading to liver transplant or death. Poor dietary habits promote disease progression, whereas a healthy diet supports liver recovery. Created in https://BioRender.com.

Over the past four decades, NAFLD has emerged as the most prevalent chronic liver disorder, affecting approximately 25% of global adult population, and has a bidirectional relationship with metabolic syndrome, contributing to its progression [67–69]. Due to its widespread prevalence, NAFLD has become the fastest-growing contributor to liver-related mortality and a major factor in end-stage liver disease, primary liver cancer, and liver transplantation, placing a considerable economic burden on healthcare systems [70, 71]. Rising incidence of NAFLD is closely linked to unhealthy lifestyle choices, particularly poor dietary habits, which also contribute to the increasing prevalence of cardiometabolic disorders and certain cancers [72–74]. Emerging evidence from animal models with altered gut microbiota and observational studies in NAFLD patients suggests a pivotal role of gut dysbiosis in disease pathogenesis [75]. Therefore, intestinal microbiota influences NAFLD development through multiple mechanisms, including modulation the of energy homeostasis, lipid and choline metabolism, ethanol production, immune regulation, and inflammatory processes [76]. Furthermore, microbiota-derived metabolites may directly impact hepatic function, thereby modulating NAFLD susceptibility [77, 78]. NAFLD is histologically classified into two forms: NAFL and nonalcoholic steatohepatitis (NASH) [79]. NAFL is characterized by fat accumulation in more than 5% of hepatocytes, affecting 15%–30% of individuals, but remains reversible with lifestyle modifications [80–82]. Without proper management, approximately 20% of NAFL cases advance to NASH, which involves steatosis, inflammation, hepatocyte ballooning, and fibrosis, but in its early stages, NASH is still reversible [83–85]. However, progression to cirrhosis occurs in 15%–25% of NASH patients, leading to irreversible liver scarring and impaired function [86, 87]. Additionally, hepatocellular carcinoma (HCC) develops in around 4%–27% of NASH cases, often resulting in liver failure, where transplantation becomes necessary [88]. Dietary habits, particularly high consumption of processed foods and sugars, contribute to disease progression, while a balanced diet and lifestyle modifications can help reverse NAFLD in its early stages (Figure 5) [89]. Notably, the modulation of the gut microbiome through probiotics, prebiotics, or symbiotic has demonstrated potential in improving liver phenotype in NAFLD patients.

#### 3.1.4. CVD

**Figure 6:**
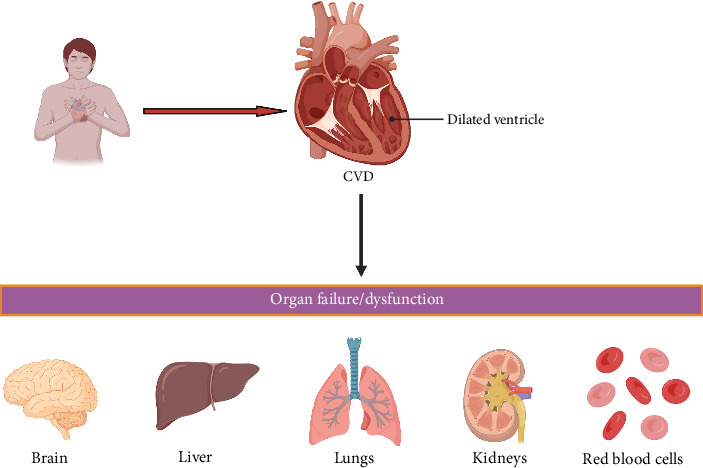
Cardiovascular disease (CVD) and its impact on multiple organ systems. The image illustrates a heart with a dilated ventricle, a hallmark of heart failure, leading to organ failure and dysfunction. The affected organs include brain, liver, lungs, kidneys, and red blood cells, highlighting the systemic consequences of CVD. Created in https://BioRender.com.

CVD remains the leading cause of morbidity and mortality worldwide, with elevated cholesterol levels recognized as a key risk factor, biomarker, and predictor due to their role in obstructing blood flow and oxygen transport [90–92]. Hypercholesterolemia is strongly associated with an increased risk of CVD, including atherosclerosis, heart failure, and hypertension, and also contributes to metabolic disorders such as diabetes, liver disease, and Alzheimer's disease, often leading to organ dysfunction (Figure 6) [93]. Emerging evidence highlights a strong connection between gut microbiota composition and cholesterol metabolism. For instance, studies using the hypercholesterolemic mice model have shown that the antibiotic-induced depletion of gut microbiota enhances cholesterol absorption and synthesis in hepatic cells, while microbiota transplantation from hypercholesterolemic human donors induces a similar dyslipidemic phenotype in recipient mice, reinforcing the causal link between microbiota composition and lipid homeostasis [92, 94, 95]. Mechanistically, specific microbial taxa such as *Lactobacillus* spp., *Akkermansia muciniphila*, and *Bacteroides* spp. influence cholesterol metabolism through bile salt hydrolase (BSH) activity, the modulation of SCFA profiles, and the activation of host signaling pathways including FXR and TGR5 [92, 96, 97]. These findings suggest that gut microbiota modulation may serve as a promising therapeutic approach for cholesterol regulation and CVD prevention. However, further research is needed to fully elucidate the pathophysiology of CVD and develop targeted therapeutic strategies for its effective management [98].

#### 3.1.5. IBD

**Figure 7:**
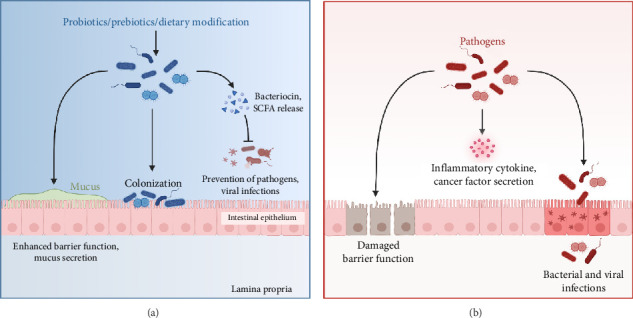
Effects of probiotic/prebiotics/dietary modification vs. pathogenic gut microbiota on intestinal barrier function. (a) The beneficial effects of probiotics, prebiotics, and dietary modifications on gut health. It enhances barrier function by promoting mucus secretion, colonizing the intestinal epithelium, and releasing antimicrobial compounds such as bacteriocins and SCFAs, which prevent pathogen colonization and viral infections. (b) The detrimental effects of pathogenic bacteria, which impair gut barrier integrity, leading to increased permeability. Pathogens promote the secretion of inflammatory cytokines and cancer-associated factors, causing barrier dysfunction and facilitating bacterial and viral infections. Created in https://BioRender.com.

IBD is a chronic, relapsing disorder characterized by persistent gastrointestinal inflammation and significant morbidity [99]. Its global incidence and prevalence have increased markedly in recent decades, posing a significant public health concerns, particularly in industrialized nations [100–102]. Pathogenesis of IBD is attributed to a complex interconnection between gut microbiota and host immune system, influenced by genetic predisposition and environmental factors [103–106]. Dysregulation of this interaction leads to aberrant immune activation, contributing to clinical and endoscopic features of disease [99, 107]. Environmental and lifestyle factors, including excessive antibiotic use, hygiene practices, and a Western diet—characterized by low fiber intake and high consumption of fat and sugar—are linked to gut microbiota dysbiosis, which may induce chronic inflammation and metabolic dysfunction [108, 109]. Disruption of the microbiota can result in the loss of essential microbial functions, leading to impaired nutrient metabolism, compromised intestinal barrier integrity, and the dysregulation of both types of innate and adaptive immune activities, ultimately affecting immune system regulation [110, 111]. SCFAs, including butyrate, acetate, and propionate, are playing a crucial role in maintaining intestinal homeostasis by promoting the growth of beneficial bacteria, modulating immune responses, and reinforcing gut barrier integrity (Figure 7). Additionally, SCFAs stimulate regulatory T cells, help to reduce inflammatory mediators, and enhance the consumption of colonic oxygen by epithelial cells, collectively supporting gut health and immune regulation [112–115]. Collectively, these mechanisms highlight the therapeutic potential of targeting gut microbiota and its metabolites for the prevention and management of IBD.

### 3.2. Therapeutic Potential of Targeting Gut Microbiome

Gut microbiome is a key regulator of metabolic homeostasis, influencing energy balance, glucose metabolism, and lipid regulation through complex host–microbe interactions [116]. Disruptions in microbial composition and function, termed dysbiosis, were responsible behind the development of common metabolic disorders, including obesity, T2D, and NAFLD [117–119]. Specific bacterial species, known as *Faecalibacterium prausnitzii* and *A. muciniphila*, play a protective role and also help to maintain gut barrier integrity and modulating inflammatory pathways [119]. Emerging research suggests that dietary interventions, along with the use of prebiotics and probiotics, can reshape the gut microbiome, enhancing SCFAs' production and reducing systemic inflammation, thereby mitigating metabolic dysfunction [120]. Furthermore, FMT has demonstrated potential in restoring microbial diversity and improving insulin sensitivity in individuals with metabolic syndrome [121]. Future therapeutic approaches should emphasize precision microbiome-based interventions tailored to individual microbial signatures, advancing personalized medicine in the management of metabolic disorders.

#### 3.2.1. *Lactobacillus* spp. as Probiotics on Metabolic Disorders

In 1989, Fuller first defined probiotics as “live microbial supplements that offer health benefits to the host by enhancing intestinal balance” [122]. Since then, extensive research has highlighted the role of gut microbiota in producing a diverse range of bioactive compounds that influence both local gut physiology and systemic health (summarized in Table 2) [160–162]. Among these, SCFAs, generated through the microbial fermentation of complex carbohydrates and proteins in colon, have been shown to support metabolic health by enhancing cholesterol utilization for bile acid synthesis, thereby reducing the risk of metabolic disorders [163]. Specific probiotic strains, such as *Lactococcus lactis* and *Bifidobacterium* spp., have demonstrated the ability to secrete insulin analogs, exerting beneficial metabolic effects in both human and animal models [164, 165]. Notably, genus *Lactobacillus*, a key member of *Firmicutes* phylum, is one of the most extensively studied probiotic groups within the gut microbiome described in Table 2 [166]. These bacteria contribute to metabolic homeostasis by regulating oxidative stress responses and modulating inflammatory pathways [166]. For instance, *Lactobacillus acidophilus* has shown antidiabetic potential by strengthening epithelial barrier function, reducing systemic inflammation, and influencing gene expression related to glucose and lipid metabolism [167]. Given these findings, probiotics represent a promising avenue for metabolic disease intervention, warranting further investigation into their therapeutic applications.

#### 3.2.2. Prebiotics as a Therapeutic Strategy for Metabolic Disorders

Prebiotics are nondigestible dietary compounds that selectively enhance the growth and metabolic activity of beneficial gut microbiota, thereby exerting significant health benefits [168]. Their role in modulating gut microbiome composition and specific functions has positioned them as promising adjuncts in the management of metabolic disorders, shown in Table 3 [185]. Emerging evidence suggests that prebiotic supplementation can improve metabolic conditions such as obesity and T2DM by promoting a favorable microbial environment that influences host metabolism [186]. Beyond metabolic regulation, prebiotics contribute to immune modulation by enhancing immune-regulatory interleukins and intestinal immunoglobulins while concurrently reducing proinflammatory cytokines [187, 188]. Additionally, they stimulate production of key SCFAs, including acetate, propionate, and butyrate, which play a critical role in maintaining gut barrier integrity and systemic metabolic balance [189]. Prebiotics have established applications in pharmaceuticals and as natural sweeteners for individuals with diabetes due to their capacity to regulate glucose metabolism [190]. Furthermore, accumulating evidence highlights their potential in CVD prevention by lowering total serum cholesterol, reducing low-density lipoprotein (LDL) cholesterol levels, and mitigating systemic inflammation [191]. Recent preclinical findings extend this evidence. Zhang et al. [192] demonstrated that arabinoxylan supplementation alleviates choline-diet–induced gut barrier dysfunction by restoring tight junction proteins (Tjp1–3, Ocln), reducing PERK pathway activation, and lowering trimethylamine (TMA) accumulation. Notably, coadministration of glycolysis inhibitors, particularly pyruvate kinase inhibitors, enhanced these protective effects, suggesting synergistic strategies for microbiota-targeted interventions. Given their multifaceted physiological benefits, further research is warranted to explore the clinical efficacy of prebiotics in disease prevention and therapeutic interventions.

#### 3.2.3. Dietary Modifications on Metabolic Disorders

Dietary modifications (summarized in Table 4) play a pivotal role in managing metabolic disorders, including metabolic syndrome, which encompasses conditions such as abdominal obesity, dyslipidemia, hypertension, and insulin resistance [197]. A diet rich in whole grains, legumes, fruits, and vegetables while minimizing the intake of refined sugars and saturated fats has been associated with significant improvements in metabolic health [198]. Notably, Mediterranean diet, characterized by an emphasis on plant-based foods, healthy fats, and lean proteins, has been widely recognized for its efficacy in regulating blood glucose levels and mitigating risk factors associated with metabolic syndrome [199]. Additionally, time-restricted eating, a type of intermittent fasting, has shown promise in enhancing insulin sensitivity and facilitating weight management [200]. When complemented with regular physical activity, these dietary interventions provide a comprehensive and sustainable approach to managing metabolic disorders and promoting overall health.

#### 3.2.4. Critical Evaluation of Mixed and Neutral Findings

Numerous studies highlight the beneficial effects of probiotics, prebiotics, dietary interventions, and FMT on metabolic disorders [201, 202], but all outcomes are not consistently positive. Some clinical trials, particularly in humans, have shown neutral or limited effects, likely due to variability in individual microbiome composition, differences in intervention dosage, and study design [203–205]. For instance, some *Lactobacillus* and *Bifidobacterium* strains show inconsistent improvements in insulin sensitivity or lipid profiles, while prebiotic supplementation sometimes fails to produce significant metabolic benefits despite promising animal data [203, 204, 206]. FMT outcomes have been variable across studies, with modest improvements in glycemic and lipid parameters but limited effects on weight reduction, and the long-term sustainability of these changes remains uncertain [207].

These mixed results underscore the complexity of host–microbiome interactions and the need for rigorous, well-powered studies that account for individual microbial signatures and intervention parameters. Considering both positive and neutral outcomes provides a balanced perspective, strengthens scientific rigor, and better understands to develop future microbiome-targeted therapeutic strategies.

### 3.3. Biomarkers for Disease Prediction and Intervention

Biomarkers have gained significant attention in predicting metabolic disorders, allowing early diagnosis and enabling targeted interventions, summarized in Table 5. These biomarkers can be molecular indicators, such as lipids, peptides, and specific genes, that reflect metabolic disturbances [219, 220]. Studies have shown that inflammatory biomarkers, such as C-reactive protein (CRP) and interleukins, play a crucial role in assessing the risk of disorders like diabetes and CVDs [221, 222]. Moreover, adipokines, such as leptin and adiponectin, are closely associated with insulin resistance and metabolic syndrome [223, 224]. Emerging research emphasizes the role of gut microbiota-derived metabolites as potential biomarkers, linking gut microbiome's impact on metabolic regulation [225]. Advances in omics technologies, including genomics and proteomics, have further enhanced the identification of novel biomarkers for more precise intervention strategies [226, 227]. By integrating these biomarkers into clinical practice, early diagnosis and personalized therapies can be effectively implemented for managing metabolic disorders.

### 3.4. Short-Chain Fatty Acids and Metabolic Disorder Intervention

**Figure 8:**
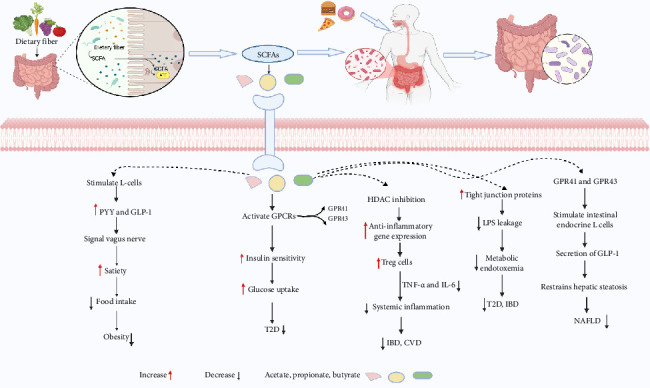
SCFA-mediated regulation of host metabolism and inflammation. Microbial fermentation of dietary fiber produces SCFAs (acetate, propionate, butyrate), which enhance insulin sensitivity, satiety, and gut barrier integrity via GPR41/43 activation and HDAC inhibition. These effects reduce food intake, systemic inflammation, and metabolic endotoxemia, mitigating obesity, T2D, NAFLD, IBD, and CVD. Created in https://BioRender.com.

Short-chain fatty acids, including acetate, propionate, and butyrate, are key microbial metabolites derived from the fermentation of dietary fiber in gut, exerting profound effects on host metabolism and immune function [228]. SCFAs stimulate enteroendocrine L cells to secrete peptide YY (PYY) and glucagon-like peptide-1 (GLP-1), which engage the vagus nerve to enhance satiety, suppress food intake, and mitigate obesity risk [229, 230]. Additionally, SCFAs activate G-protein–coupled receptors (GPCRs), notably GPR41 and GPR43, thereby improving insulin sensitivity and glucose homeostasis, offering protection against T2D [231, 232]. These metabolites further reinforce intestinal barrier integrity by upregulating tight junction proteins, limiting lipopolysaccharide (LPS) translocation and metabolic endotoxemia, mechanisms implicated in T2D and IBD [15, 233, 234]. SCFAs also function as histone deacetylase (HDAC) inhibitors, promoting anti-inflammatory gene expression and inducing regulatory T (Treg) cell differentiation, leading to reduced systemic inflammation via the suppression of TNF-α and IL-6, thereby conferring protection against IBD and CVD [15, 231, 235]. Furthermore, SCFAs are able to inhibit HDACs that helps to regulate the inflammatory response during NAFLD. SCFAs interact with GPR41 and GPR43 receptors to stimulate the secretion of GLP-1 by intestinal endocrine L cells, helping to reduce hepatic steatosis and activate brown adipose tissue, thereby improving NAFLD [236]. These insights underscore the critical role of SCFAs as metabolic and immunological mediators, linking gut microbiota function to host health and disease susceptibility (Figure 8).

### 3.5. Limitations

While this review provides comprehensive coverage of current evidence, certain limitations exist. Heterogeneity among study designs, variations in intervention types, and inconsistent outcome measurements limit direct comparisons. The predominance of animal studies reduces the generalizability to human populations, highlighting the need for more standardized and large-scale human clinical trials to validate the findings of microbiome-based interventions. Some studies and clinical trials reported neutral or limited effects, underscoring that positive findings are not always consistently replicated in humans (for a more detailed discussion, see Section 3.2.4). Furthermore, a substantial proportion of the literature focuses on positive outcomes, which may reflect publication bias and overestimation of therapeutic potential. Additionally, the absence of PROSPERO or equivalent registration, despite adherence to PRISMA guidelines, limits methodological transparency, making it harder to assess potential biases and reproduce the review reliably.

## 4. Conclusion and Perspectives

Gut microbiota plays an important role in the pathophysiology of common metabolic disorders, including obesity, T2DM, CVD, NAFLD, and IBD. Emerging evidence highlights the therapeutic potential of modulating gut microbiota composition through probiotics, prebiotics, dietary modifications, and microbial metabolites. These strategies have demonstrated promising outcomes in restoring microbial balance, improving metabolic markers, and reducing inflammation associated with metabolic disorders. However, current research is largely limited to preclinical and small-scale human studies, necessitating further large-scale, well-designed clinical trials to validate efficacy, safety, and long-term impacts. Additionally, personalized microbiome-based interventions hold promise for precision medicine approaches but require deeper mechanistic insights and advancements in microbiome profiling technologies. Future research should focus on elucidating host–microbiota interactions, identifying novel therapeutic targets, and integrating multi-omics approaches to enhance clinical translation. This review not only consolidates recent advancements in microbiota-targeted therapy but also highlights key research gaps, encouraging the integration of microbiome science into the development of future precision medicine strategies for metabolic disorders. By leveraging gut microbiota modulation as a therapeutic strategy, we may pave the way for innovative, microbiome-driven interventions to combat metabolic disorders effectively.

## Figures and Tables

**Table 1:** Comparative summary of microbiome changes in various metabolic disorders.

Metabolic disorder	Key microbiome alterations	Dominant taxa	Clinical insight	References
Obesity	Altered *Firmicutes*/*Bacteroidetes* ratio	↑*Firmicutes*, ↓*Bacteroidetes*	Enhanced energy harvest⟶ weight gain	[23, 24]
Type 2 diabetes	Dysbiosis, decreased SCFA-producing bacteria	↑*Bacteroidetes*, ↑*Firmicute*, ↓*Clostridium*	Link to insulin resistance and bile acid dysregulation	[25–29]
Nonalcoholic fatty liver disease	Dysbiosis, overgrowth of ethanol-producing bacteria	↑*Bacteroides*, ↑*Ruminococcus,* ↓*Prevotella*	Associated with hepatic steatosis and inflammation	[30, 31]
Cardiovascular disease	Dysbiosis with increased TMAO producers	↑*Prevotella*, ↑*Bacteroides*	TMAO promotes atherosclerosis predictive of cardiac risk	[32, 33]
Inflammatory bowel disease	Reduced diversity, increased pathogenic bacteria, decreased beneficial bacteria	↓*Bacteroidetes,* ↑*Proteobacteria*, ↑*Firmicute*	Imbalanced gut microbiota⟶ chronic inflammation, impact treatment response	[34–36]

*Note:* While consistent patterns were observed across disorders (e.g., an increase in *Firmicutes* in obesity and diabetes), some findings such as the rise in *Bacteroidetes* in both obesity and T2D appear contradictory to earlier studies. These variations may reflect dietary, genetic, or methodological differences across the included studies.

**Table 2:** Roles of *Lactobacillus* spp. as a probiotic in mitigating metabolic disorders: mechanisms and evidence.

Species	Functional effects	Targeted disease/condition	Clinical/preclinical evidence	References
*Lactobacillus curvatus* HY7601	↓Insulin, leptin, total cholesterol; ↓TNFα, IL6, IL1β, and MCP1; ↑PGC1α, CPT1, CPT2, ACOX1	Obesity	Reduce diet-induced obesity	[123]
*Lactobacillus plantarum* KY1032				
*Lactobacillus casei* strain Shirota (LcS)	↓Leptin and glucose; ↑lipoprotein and adiponectin; ↓IL-6		Enhances body weight management	[124]
Lactobacillus sakei Probio65	↓Triglyceride deposition; ↓PAI-1, leptin, TNF-α, STAMP2, F4/80, resistin, and MCP-1; ↑GLUT4 and adiponectin		Effective in mitigating obesity and improving adipocyte function	[125]
Lactobacillus plantarum Probio-093				
*Lactobacillus acidophilus* CICC 6075	↑Histidine biosynthesis, ↓NF-κB pathway		Promising probiotic candidate for obesity prevention	[126]
*Lactobacillus plantarum K50 (LPK)*	↓Cholesterol, triglyceride, leptin		Reductions in total fat mass or in body weight	[127]
*Lactobacillus reuteri* J1	↓FXR; ↑GPCRs-5; ↓FXR/FGF15, ↑FXR/SHP; ↓triglyceride accumulation; ↑UCP1		Prevents weight gain, reduces fat mass, relieves dyslipidemia, and improves glucose homeostasis and insulin sensitivity	[128]
*Lactobacillus paracasei N1115*	↓Triglyceride, TC, ↓LDL; ↓fatty acid, ↓IL-1 β, ↓TLR-4		Reduces weight gain and liver fat accumulation	[129]
*Lactobacillus paracasei* L*9*	↓Cholesterol, triglyceride, LDL; ↓proinflammatory cytokine, lipid synthesis–related genes		Natural alternative to attenuate obesity	[130]
*Lactobacillus fermentum* CQPC07	↓TC, LDL-C, TG, ↑HDL-C; ↓IL-1β, ↓TNF-α, ↓IL-6, ↓IFN-γ; ↑IL-10; ↓IL-4; ↓ALT, AST, ALP		Potential probiotic for prevention and treatment of obesity	[131]
*Lactobacillus rhamnosus LRH05 (LRH05)*	↓Body weight, WAT; ↑hepatic steatosis and glucose intolerance; regulates the Mogat1, Igf-1, Mcp-1, and F4/80; ↓macrophage infiltration in WAT		Potential probiotic strain for obesity prevention	[132]
*Lactobacillus plantarum* strain K21	↓Leptin, cholesterol, triglyceride, PPAR-γ; modulate *Lactobacillus* spp., *Bifidobacterium* spp., *Clostridium perfringens*		Protects from HFD-induced obesity	[133]
*Lactobacillus plantarum* KC28	↑PGC1-α, CPT1-α; PPAR-γ; FAS; ↑*Desulfovibrionaceae*, *Lactobacillaceae*		Protective effects against diet-induced obesity mice	[134]
*Lactobacillus rhamnosus* 4B15	↓Itgax, Emr1, TNF-α; Ucp-1; ↓SREBP, chREBP		Provide symbiotic effects against obesity	[135]
*Lactobacillus plantarum* FRT10	↓TGs, ALT; ↑*Butyricicoccus*, *Butyricimonas*, *Intestinimonas*, *Odoribacter*, and *Alistipes*; ↓*Desulfovibrionaceae*, *Roseburia*, and *Lachnoclostridium*, *Lactobacillus*, *Bifidobacterium*, and *Akkermansia*; ↑PPARα, CPT1α; ↓SREBP-1, DGAT1		Single probiotic for reducing HFD-induced obesity.	[136]
*Lactobacillus plantarum* 23-1	↑TLR4/NF-κB, ZO-1, occludin, claudin-1, Muc2; ↓inflammation		Decreasing obesity and offering a conceptual framework for creating novel functional foods	[137]

Lactobacillus casei K11	↓Blood glucose and ameliorated insulin resistance; lipid metabolism, oxidative stress; ↑FFAR2, GLP-1, SCFAs	T2D	Modified SCFA–FFAR2–GLP-1 pathway to improve T2DM	[138]
Lactobacillus casei CCFM419	↓Glucose, glucose intolerance, insulin resistance; TNF-α, IL-6; ↑GLP-1; *Bacteroidetes*; ↓*Firmicutes*; ↑SCFA-producing bacteria		Altered gut flora-SCFA-inflammation pathway to improve T2D	[139]
*Lactobacillus plantarum* HAC01	↓Blood glucose and HbA1c; ↑glucose tolerance and HOMA-IR; ↑insulin-positive β-cell and ↓mRNA expression; ↑phosphorylation of AMPK and Akt; ↑*Akkermansiaceae* and ↑SCFAs		Alleviate hyperglycemia and T2DM by regulating glucose metabolism	[140]
*Lactobacillus plantarum-*pMG36e-GLP-1	↑β-cell proliferation, insulin secretion; ↓inflammatory reaction; regulated gut microbiota		Giving a potential approach to T2DM treatment	[141]
*Lactobacillus* G15 and Q14	↑SCFA-producing bacteria, acetate, butyrate, GPR43; ↓LPS, inflammatory factors		Antidiabetic efficacy through gut microbiome interactions	[142]
*Lactobacillus gasseri* CKCC1913	↑Glucose tolerance, HDL; ↓blood glucose, TNF-α, IL-6; ↑*Parabacteroides merdae*		Potential probiotics effects to treat diabetes	[143]
*Lactobacillus rhamnosus* ZJUIDS07	↑SCFAs; ↓*Staphylococcus*, *Corynebacterium*, *Odoribacte*; ↑*Muribaculaceae norank, Dubosiella, Clostridium sensu stricto 1, Lactobacillus, Faecalibaculum*		Potential probiotic approach for future gut microbiome-based T2D treatment	[144]
*Lactobacillus reuteri* TISTR 2736	↓IRS1, IRS1^Ser307^; ↑PI3K, AKT^Ser473^; ↓NF-κB, IL-6, TNF-α		Reduced hyperglycemia and enhanced metabolism of carbohydrates	[144]

*Lactobacillus sakei* MJM60958	↓Liver weight, ALT, AST, TG, BUN, UA; steatosis, leptin, IL; ↑adiponectin; ↓FAS, ACC, SREBP-1; ↑PPARα, CPT1A; *Verrucomicrobia*, *Firmicutes*	NAFLD	Shows potential as a probiotic for preventing and treating NAFLD	[145]
*Lactobacillus plantarum* NCU116	↓Fat accumulation; endotoxin, proinflammatory cytokines; ↓lipogenesis; ↑lipolysis, FA		Helps to restore liver function	[146]
*Lactobacillus salivarius SNK-6*	↓Liver fat, TG, ALT, AST; ↑MBOAT2, SCFAs		Liver damage alleviated via miR-130a-5p/MBOAT2 signaling pathway	[147]

*Lactobacillus plantarum* DMDL 9010	↓Cholesterol, LDL-C, atherosclerosis index; ↑bile acids	CVD	Potential probiotic for hypercholesterolemic prevention and therapy	[148]
*Lactobacillus plantarum* GLP3	↓TMAO; ↑*Lactobacillus* spp.		Helps to reduce cardiovascular disease	[149]
*Lactobacillus rhamnosus* SKG34	↓Cholesterol, TG, HDL-c; HDL-c; ↑lactic acid bacteria		Helps to improve blood lipid profile	[150]
*Lactobacillus rhamnosus* FBB42				
*Lactobacillus reuteri* GMNL-263	↓NF-κB, COX-2, CTGF, SP1		Given protective effects against cardiac injury	[151]
*Lactobacillus fermentum* MJM60397	↓TC, LDL; ↑Bile acids, LDLR gene		Contribute to reduce cholesterol levels in serum	[152]

*Lactobacillus plantarum* HNU082	↑SCFAs; ↓ICAM-1, VCAM, NF-kB; ↑goblet cells, mucin 2, ZO-1, IL-10, TGF-β1, TGF-β2	IBD	Potential gut microbiota regulator to treat UC	[153]
*Lactobacillus johnsonii* GLJ001	↓mRNA expression, TNF-α, Il-1β, ↓M1 macrophage; ↑SCFAs-producing bacteria		Recognized as a targeted approach to prevent IBD	[154]
*Lactobacillus gasseri G098*	↓IL-6, IL-1β, TNF-α; ↑IL-13; *Firmicutes*, *Lachnoclostridium*; ↓*Bacteroidetes*		Mitigate colitis through modulating host immunity and gut microbiome	[155]
*Lactobacillus rhamnosus* ZFM231	↑Acetic acid, propionic acid, butyric acid, anti-inflammatory factor, TGF-α; ↓TNF -α		Significantly alleviate symptoms of DSS-induced IBD	[156]
*Lactobacillus paracasei* MSMC39-1	↓TNF-α; ↑*Lactobacillus*		Exhibit an anti-inflammatory impact in the colon and altered the gastrointestinal flora	[157]
*Lactobacillus gasseri* ATCC33323	↓IL-1β, IL-6, TNFα; ↑E-cadherin, MUC2, ZO-1, Claudin 1, β-catenin, p120-catenin, occluding		Provide alternate treatment for IBD, notably food supplementation	[158]
*Lactobacillus plantarum* SS18-50	↓TNF-α, COX-2, IL-10, IL-6, MPO, MDA, ↑SOD, somatostatin		Help to inhibit the development of colitis	[159]

*Note:* Several *Lactobacillus* strains demonstrated consistent anti-obesity effects across preclinical studies, such as reductions in leptin, triglycerides, and proinflammatory cytokines. Notably, *L. reuteri* J1 showed a multipathway mechanism involving FXR and UCP1 modulation. However, some strains such as *L. plantarum* KY1032 were listed without mechanistic data, which limits comparative interpretation.

**Table 3:** Prebiotics and their role in managing metabolic disorders through gut microbiome interaction.

Prebiotic	Intervention/study design	Dose/concentration	Study duration	Mechanism of action	Clinical evidence	Target disease	References
Inulin	Double-blind, randomized, placebo-controlled crossover	0.5 g was U-^13^C-inulin/24 g of oral inulin	5 days	↑Fat oxidation, plasma glucose; ↓insulin, ↑SCFA	May improve human metabolism	Obesity	[169]
Fructo-oligosaccharides (FOS)	In vitro culturing	—	24 h	↑SCFA; ↓alpha diversity and shifted communities	Promote gut health	Obesity, IBD	[170]
Galacto-oligosaccharides (GOS)	In vivo	—	3 weeks	↓*Ruminococcaceae*, *Oscillibacter*; ↓fatty acid, triglyceride; ↑*Alloprevotella*, *Bacteroides*, *Parasutterella*	Improve lipid and glucose metabolism	Obesity, diabetes	[171]
Arabinoxylans	—	(10% w/w)	4 weeks	↑*Bacteroides*-*Prevotella* spp., *Roseburia* spp., cecal bifidobacteria	Lipid-lowering effects and anti-obesity effects	Obesity	[172]
Yeast β-glucan	In vivo	100 μL (or 100 μg) o	30 days	↑IL-10, IL-17, and IL-21; ↓TNF-α, suppressed insulitis	Enhance gut microbiota and immune functions	T1D	[173]
Pectin	In vitro	—	—	↑*Eubacterium eligens*	Rebalances microbiota toward an anti-inflammatory profile	IBD	[174]
Xylo-oligosaccharides (XOS)	Randomized, placebo-controlled crossover	2 g/day	8 week	*↓Howardella, Enterorhabdus*, *Slackia*; ↑*Blautia hydrogenotrophica*; ↓OGTT 2-h insulin levels	Reversing gut microbiota alterations	T2DM	[174]
Polydextrose	In vivo	Western diet (WD) ±75 mg twice daily	14 days	↓triglyceride, cholesterol, Dgat1, Cd36, Fiaf; ↑Fxr, *Allobaculum*, *Bifidobacterium, Coriobacteriaceae* taxa	Systemic metabolic improvements via gut microbiota regulation	Obesity	[175]
Isomalto-oligosaccharides (IMOs) with cranberry extract (CRX)	In vivo	200 mg/kg,	12 weeks	↑Butyrate, beneficial bacterial abundance; prevent tissue inflammation, glucose intolerance	Normalization of metabolic alterations	Obesity	[176]
Manno-oligosaccharides	In vivo	300–1200 mg per kg b.w. per d	8 weeks	↓Glucose, endotoxemia; ↑*Bifidobacteria*; ↓*Shigella*, regulated AKT/IRS/AMPK signaling pathway	Modulate metabolic inflammation	Diabetes	[177]
Chicory root	In vivo and in vitro		5-week	Modified abundance of *Alloprevotella*, *Blautia*, *Alistipes*, *Oscillibacter*; ↑CCK and GLP-1 satiety hormones	Appetite regulation	Obesity	[178]
Raffinose	In vivo study methos	100, 200, or 400 mg/kg	21 days	Control IL-2, IL-6, IL-1β, and TNF-α; ↓TLR4–MyD88–NF-κB pathway	Protective effect in colitis by modulating gut microbiota	IBD	[179]
Fucoidan	In vivo study methos	200 mg/kg	Sixteenth week	↑*Akkermansia muciniphila*, *Alloprevotella*, *Blautia*, *Bacteroides*	Reduces MetS via gut microbiota modulation.	Inflammation, obesity and diabetes	[180]
Alginate oligosaccharides	In vivo study methos	5 g AO per 100 g mice	10 weeks	↓TG, LDL-C, lipogenesis genes, glucose; ↑serum insulin; ↓IL1β, CD11c; ↑*Akkermansia muciniphila, Lactobacillus reuteri, Lactobacillus gasseri;* ↑acetic acid, propionic acid, butyric acid; ↓endotoxin	Enhances lipid metabolism and reduces inflammation	Obesity	[181]
Dextrins	In vitro method	—	24 h	↑*Actinobacteria*, *Bacteroidetes*; ↓*Firmicutes*	Supports weight management by modulating gut microbiome	Obesity	[182]
Chitosan oligosaccharides	In vivo study methos	—	56 days	↑*Clostridium paraputrificum*, *Clostridium ramosum*; ↓*Clostridium cocleatum*; ↓liver fat; normalize glucose and insulin	Modulating intestinal microbiota	Obesity	[183]
Konjac glucomannan	In vivo study methos	80 mg/kg b.w	28 days	↓TC, TG, VLDL, LDL; ↑insulin levels, regulate PPAR-γ, *p*-SREBP-1C, produce SCFAs	Inhibiting lipid absorption and regulates microbiome	T2DM	[184]

*Note:* Most prebiotics improved metabolic health by enriching beneficial microbes (e.g., *Bifidobacterium* and *Akkermansia*), increasing SCFA levels, and modulating inflammation and metabolism. Some unexpected reductions in microbial diversity suggest that responses may vary by host. Standardized clinical trials are needed to confirm long-term benefits.

**Table 4:** Dietary modifications and their beneficial effects on metabolic disorders.

Dietary modification	Mechanism of action	Targeted disease/condition	Clinical/preclinical evidence	References
High-fiber diet	↑Glucose homeostasis; altered-serum metabolome, psychiatric comorbidities and systemic inflammation; ↑beneficial bacteria	T2D	Improves metabolism and emotional health via microbiome modulation	[193]
Polyphenol-rich diet	↓Blood glucose; ↑insulin secretion and sensitivity	T2D, CVD	Enhances early insulin response and reduces postprandial glucose	[194]
Mediterranean diet	↓hs-CRP, IL-6, IL-7, IL-18; ↓insulin resistance	Obesity, T2D, CVD	Reduces metabolic syndrome and lower cardiovascular risk	[195]
Ketogenic diet	↓TG and total cholesterol; ↑HDL levels	T2DM, NAFLD, and CVD	Improves lipid profile and reduces insulin resistance	[196]

*Note:* Dietary interventions such as high-fiber, polyphenol-rich, Mediterranean, and ketogenic diets exhibit positive effects on glucose regulation, lipid metabolism, and systemic inflammation, primarily via gut microbiota modulation and metabolic reprogramming. However, individual variability and potential adverse effects (e.g., sustainability issues with ketogenic diets) warrant further long-term and personalized clinical studies.

**Table 5:** Biomarkers for disease prediction and intervention: expanded insights from gut microbiome and metabolic disorders.

Biomarker	Disease/condition	Biomarker function	Findings/outcomes	References
Short-chain fatty acids	Obesity, Type 2 diabetes	Modulate insulin sensitivity and inflammation via microbial fermentation of fiber	Elevated SCFAs like butyrate are associated with improved insulin sensitivity and weight regulation	[208]
Fecal microbiota profile	Obesity, metabolic syndrome	Predictive biomarker based on gut microbiota composition	Dysbiosis, particularly a reduction in *Firmicutes* to *Bacteroidetes* ratio, is associated with obesity and insulin resistance	[209]
Bile acids	Obesity, Type 2 diabetes, nonalcoholic fatty liver disease	Modulation of lipid metabolism, glucose homeostasis, and inflammation	Altered bile acid profiles, particularly an increase in primary bile acids, may indicate poor metabolic health and liver dysfunction	[210]
LPS (lipopolysaccharide)	Obesity	Marker of low-grade systemic inflammation due to gut permeability	Increased plasma LBP levels are associated with BMI, insulin resistance, and systemic inflammation in overweight/obese adolescents	[211]
Trimethylamine-N-oxide (TMAO)	Atherosclerosis, cardiovascular disease	Indicate gut-derived TMAO production via γ-butyrobetaine (γBB)	Higher plasma γBB levels are associated with increased cardiovascular risk	[212]
Gut microbiome	Type 1 diabetes, Crohn's disease	Microbiome shifts like ↓ *Akkermansia muciniphila* as early biomarkers for disease onset	Sub-clinical changes in the gut microbiome, including reduced *A. muciniphila* and oxidative stress responses, may precede T2D onset	[213]
Aryl hydrocarbon receptor (AHR)	Gestational diabetes mellitus	Biomarker for persistent organic pollutants (POPs) and risk of gestational diabetes mellitus (GDM) via AhRT activity	Higher serum AhRT activity and lower ATP concentrations are associated with increased GDM risk and correlate with glucose levels during GDM screening	[214]
Circulating miRNAs (micro-RNAs)	Metabolic syndrome, obesity	Reflect adipokines related metabolic dysfunction	Altered miRNA expression correlates with BMI, adipokines, and metabolic syndrome-related biomarkers, including insulin and lipids	[215]
Fecal calprotectin	Inflammatory bowel disease	Marker of neutrophilic intestinal inflammation	Calprotectin correlates with histological inflammation, distinguishes IBD from irritable bowel syndrome, and aids in predicting relapses and monitoring treatment responses in IBD	[216]
Ceramides	Cardiovascular disease	Reflect lipid-mediated cardiovascular risk	Circulating ceramides decreased, while ceramide ratios and cardiac event risk scores increased in older adults after 5 days of bed rest	[217]
Branched-chain amino acids (BCAAs)	Obesity, insulin resistance	Regulate muscle metabolism and contribute to insulin resistance	Increased BCAA levels are associated with obesity and impaired insulin signaling	[218]

*Note:* The biomarkers highlighted here provide mechanistic insights into gut-microbiota–related metabolic dysfunctions, including obesity, diabetes, and cardiovascular disease. While many show strong correlations (e.g., SCFAs, calprotectin, and LPS), causal relationships remain to be fully established. Interindividual variability, dietary influences, and methodological differences pose challenges, emphasizing the need for standardized longitudinal studies.

## Data Availability

All data generated or analyzed during this study are included in this published article. Additional materials such as the PRISMA checklist and extracted data tables are available upon reasonable request.

## Supporting Information

Supporting Information 1 Supporting File 1: This file contains the completed PRISMA 2020 checklist, which details how each reporting item has been addressed throughout the manuscript. It provides a structured summary of adherence to the PRISMA guidelines for systematic reviews.

Supporting Information 2 Supporting File 2: This table presents the comprehensive dataset extracted from the included studies, including study characteristics, methodological details, and summary of main findings. It supports the analyses and interpretations discussed in the main text.
